# Associations of functional health literacy with socioeconomic and demographic status among Filipinos

**DOI:** 10.1186/s12889-022-14602-x

**Published:** 2022-11-28

**Authors:** Lourdes Marie S. Tejero, Kathryn Lizbeth L. Siongco, Paul Adrian V. Pinlac, Kim Carmela D. Co, Ma. Carmen C. Tolabing

**Affiliations:** 1 College of Nursing, University of the Philippines Manila, Manila, Philippines; 2 Technology Transfer and Business Development Office, the University of the Philippines Manila, Padre Faura Street, Ermita 1000, Manila, Philippines; 3 College of Public Health, University of the Philippines Manila, Manila, Philippines

**Keywords:** Functional Health Literacy, Socioeconomic Position, Population Survey, National Health Literacy, Prose Literacy, Health Information

## Abstract

**Background:**

Health literacy determines an individual’s decision-making process in providing judgment concerning appropriate healthcare. Considering the various purposes of literacy for people, functional health literacy (FHL) was identified as a type of literacy that is measured by the application of literacy skills to health-related materials as a result of health education. The objective of this study was to identify the possible socio-demographic correlates of FHL of Filipinos.

**Methods:**

A cross-sectional survey was conducted among 2,303 Filipinos aged 15 to 70 years old in 2018–2019 utilizing the National Health Literacy Survey. Functional health literacy was determined using the FHL-5 Test which measured prose, document, and numeracy skills. Descriptive analysis presented socio-demographic characteristics and level of FHL. Log-binomial regression was used to estimate associations of FHL adequacy with socio-demographic characteristics.

**Results:**

Overall, the study involved 1,997 (86.7%) qualified participants. Most of them demonstrated prose literacy skills and had adequate FHL. Adjusted regression analyses showed that participants with a college-level education (Adjusted prevalence ratio [APR]: 1.43, 95% confidence interval [95% CI] 1.27–1.60), categorized under domestic service occupation (APR 1.19, 95% CI: 1.03–1.37), and those residing in urban areas (APR: 1.14, 95% CI 1.06–1.24) were significantly more prevalent in exhibiting FHL adequacy. In contrast, male participants and individuals aged 60 years or above were less likely to demonstrate FHL adequacy.

**Conclusions:**

The study presents a baseline assessment of the functional health literacy level, measured using the FHL-5 Test, of adults in the Philippines. Majority of the participants demonstrated FHL adequacy, with relatively higher scores in prose than numeracy literacy. The measurement of FHL adequacy can inform policies on methods of health education and communication, emphasizing the need to stratify the audience based on socio-demographic characteristics and adapt the content and design of informational materials for population-based health educational programs.

## Background

People live on a day-to-day basis making decisions that in one way or another, directly or indirectly, affect their health. Only a few of these decisions are made with health provider consultations, and much more often, the complicated choices are dealt with by individuals (or their caregivers) on their own. From recognizing symptoms, seeking information in the internet, “consulting” relatives and friends, self-diagnosing and self-medicating, until finally reaching the point of getting a clinic appointment: all these steps require that a person is able to locate, digest and use information for improving if not maintaining one’s health [1]. This decision-making process requires *health literacy*, a competency allowing the access, appraisal, and application of health information when making judgment concerning healthcare, disease prevention, and health promotion. The concept is at least half a century old. From the time the compound word health literacy was coined in 1974, various definitions of the term have come about, and there was a lack of consensus as to what it is exactly and how it is measured. Even with its inclusion in the World Health Organization's Health Promotion Glossary [2], the idea remained rather complex and confusing.

Foremost of the challenges is delineating between health literacy from its root word ‘literacy’. Experts argue that while both refer to a set of skills or a learning process, literacy by itself is a factor that contributes to (or hinders) health literacy. Nevertheless, the various aspects of literacy have often been adapted to describe health literacy. The National Assessment of Adult Literacy (NAAL), a U.S. survey among 19,000 adults in 2003, measured three skills (or scales) on literacy with print materials: prose, document, and quantitative. Prose literacy gauges one’s ability to search, understand, and utilize information after reading narrative text. Measures for prose literacy assess the reading skills at the pre-university secondary education level [3]. Document literacy tests the competency to appraise and use information from “noncontinuous texts” such as forms, maps, tables, and the like. Quantitative literacy (or numeracy) assesses proficiency in computation for practical applications [4–6]. Numeracy in health “refers to a patient’s ability to understand and use quantitative information to make informed clinical decisions [7]. These skills form the bases for the functional health literacy aspect of existing survey instruments, such as the Test of Functional Health Literacy in Adults (TOFHLA) and the Newest Vital Sign (NVS) [8, 9].

Meanwhile, some have gone to the extent of differentiating it with other related terms like 'medical literacy', that is more disputably applicable within health facilities. In the fifth iteration of the Healthy People initiative of the U.S. Department of Health and Human Resources, the Health Literacy Workgroup endorsed an expanded definition where health literacy was described in two distinct entities: personal (individual) and organizational. Personal health literacy essentially maintains the definition of one’s ability to obtain, comprehend, and utilize health information and services to inform decision-making, but with the recognition of the organizations’ role in enhancing equity in health literacy [10–12]. Regardless of the long history of continuing debatable semantics, the significance of developing this capacity in an individual to take charge of his or her own health has only been highlighted throughout the years.

One of the leading researchers in the subject matter, Don Nutbeam, proposed a prototypical model that divides health literacy into three levels, according to educational goals: (1) functional health literacy, which is anchored on successful health education and transmission of information; (2) interactive health literacy, which targets personal skills development; and (3) critical health literacy, which is oriented to individual and social action. Being the most basic, functional health literacy (FHL) seems to also be the most measurable of the three, where the outcome is tested with the communication of health risks and health services utilization, and determined by an improvement in knowledge and compliance at the individual level. The same term has been described to mean adequate functional capacity in healthcare settings gauged using tools on basic reading and writing skills that will enable a patient to access written health-related materials [13–15]. Numeracy, which accounts for the facility for carrying out fundamental mathematical concepts, has likewise been attached to [functional] health literacy, although evidence on its impact on health outcomes or service utilization remains inconclusive [16, 17].

Akin to the broader health literacy, the lack of or inadequacy in FHL is known to be linked with poorer health-related choices and actions, lower service uptake, suboptimal health status, and increased morbidity and mortality. Numerous attempts have been made in the last few decades to assess health literacy and FHL [18–20], although a standardized tool is yet to be designed. The use of standardized tools and validation of existing tools will likewise allow the comparison of health literacy and its determinants across populations and settings [21–25].

One of the more novel screening tools for functional health literacy is the Newest Vital Sign or NVS, introduced by Barry Weiss and colleagues in 2005 under the Pfizer Clear Health Communication Initiative. The three-minute instrument presents a nutrition label and asks only six questions where getting more than two items incorrectly indicates possible limited literacy. Throughout its development, a series of prospective scenarios and questions have been tested in more than 1,000 respondents; the final short form of tool has been found to be reliable (Cronbach α > 0.76) and accurate against the more established TOFHLA (Area under the Curve [AUC] = 0.88) [9].

The challenge of having a variety of psychometric and qualitative tools in ascertaining health literacy does not and should not preclude the conduct of health literacy and FHL measurement [3, 7, 26]. As the World Health Organization (WHO) Regional Office for South-East Asia puts it in its health literacy toolkit for low- and middle-income countries, “measurement of the health literacy strengths and limitations of communities allows strategic design and delivery of interventions that address health inequities, improve health outcomes and strengthen health systems” [27]. Moreover, as with the example of other large-scale population-based cross-sectional studies on adult health literacy, these surveys do not just end with assessing the extent of health literacy, but also identify demographic and behavioral characteristics that are its potential determinants [5, 28, 29]. The Philippines remains to be one of the developing countries that can benefit from a nationwide survey on FHL, whose results can inform policies and programs on health information dissemination, capacity-building, advocacy and social mobilization efforts towards empowerment of Filipinos in health decision-making. The study aims to identify the possible socio-demographic correlates of FHL of Filipinos.

## Methods

### Study design and setting

This study was conducted as part of the national health literacy survey, a cross-sectional study conducted in 2018–2019 with the general objective of describing the general health literacy status of Filipinos aged 15–70 years old. A multi-stage sampling strategy was conducted to select 2,303 participants by probability proportional to size sampling. The whole country was first stratified into four subnational groups: Luzon, Visayas, Mindanao, and Metro Manila. Systematic sampling was done to select provinces, cities, barangays, and households from each stratum. This sample size was sufficient to generate national and subnational estimates for health literacy level, based on the minimum sample size calculated. Details of the sampling strategy and sample size estimation have been previously described [30]. Excluded were those with cognitive impairment based on the Mini-Cog® test result. The Mini-Cog is a screening test for cognitive impairment. This was administered to each prospective participant during the recruitment. If the test result revealed cognitive impairment, the individual was excluded from the study.

Survey instrument.

Data were collected through Computer-Assisted Personal Interview (CAPI) by trained interviewers. Questions included socio-demographic and healthcare characteristics, healthcare utilization, and healthcare literacy. The socio-demographic characteristics included in the study were sex, age, island of residence, place of residence, civil status, education, occupation, religion, income, and type of health insurance. The type of health insurance was categorized as either public (National Health Insurance) or private health insurance. For measuring functional health literacy, the FHL-5 Test was used. This instrument consisted of the following questions in the FHL-5 Test pertaining to the contents of an information, education, communication (IEC) material with immunization schedule created by the Philippine Department of Health:

- 1. Which vaccines may be given to infants immediately after birth?
- 2. How many types of vaccines should have been received by a child who is one year old?
- 3. In total, how many doses of all the recommended vaccines should be given to infants?
- 4. Which vaccines protect against pneumonia?
- 5. Assume that you have a child who was born on January 1, 2017, when should he/she receive the first dose of the measles-mumps-rubella (MMR) vaccine?

These questions were constructed to test the FHL skills of document literacy, prose literacy, and numeracy domains [31] among Filipinos. Items 1 (“Which vaccines may be given to infants immediately after birth?”) and 4 (“Which vaccines protect against pneumonia?”) describe the prose literacy domain while items 2 (“How many types of vaccines should have been received by a child who is one year old?”), 3 (“In total, how many doses of all the recommended vaccines should be given to infants?”), and 5 (“Assume that you have a child who was born on January 1, 2017, when should he/she receive the first dose of the measles-mumps-rubella (MMR) vaccine?”) pertain to skills under the numeracy domain. The contents of the IEC material with immunization schedule were presented in a tabular format which entailed the participants to exhibit document literacy skills to be able to read and understand the information in the table and correctly answer the given items.

The questions were developed in consultation with health literacy and public health specialists, and were constructed to measure skills on prose literacy, document literacy, and numeracy, patterned after the Newest Vital Sign (NVS) tool. The FHL-5 Test was scored according to the questions in the IEC material provided to the participants. Items on the FHL-5 Test were scored by giving 1 point for each correct answer. The validity of FHL-5 test was assessed by computing its sensitivity and specificity. Sensitivity is its ability to label correctly as adequate FHL those who truly have adequate FHL using the NVS instrument as gold standard. Specificity is the ability of the test to label correctly as inadequate FHL those who truly have inadequate FHL using the same gold standard. Reliability of the test was measured using Cronbach’s alpha. The tool was validated using criterion-based validation measures during the pilot-testing phase of the national health literacy survey, using the NVS as the gold-standard. Compared to functional health literacy classifications using the NVS, a cut-off score of 60% had a sensitivity of 60.9%, specificity of 97.2% in identifying inadequate FHL. For this study, a score of ≥ 60% (≥ 3 out of 5) in the FHL-5 test was considered adequate FHL; otherwise, inadequate. The instrument was reported to have a Cronbach’s alpha of 0.76 [32].

### Study participants

Participants aged 15–70 years old were included in the study. They were provided with an IEC containing information of the immunization schedule which they were allowed to read without a time limit. After reading, they had to answer the five questions with instructions to refer to the material while answering the questions. Respondents who were unable to read the health education material due to illiteracy or poor vision were unable to answer the functional health literacy questions and were excluded from this study. Those who had prior knowledge about vaccination were likewise excluded. Prior knowledge was established before the administration of the instrument. Exclusion criteria were illiteracy, self-reported poor vision, prior knowledge about vaccination schedule, and cognitive impairment based on the result of the Mini-Cog © test that was administered during participant recruitment.

### Data analysis

Descriptive analysis was performed to summarize the FHL adequacy, and socio-demographic and economic characteristics of participants using frequencies and proportions. Log-binomial regression was performed to measure the association between functional health literacy and socio-demographic characteristics. Prevalence ratios (PR) assessed the strength of the associations and were adjusted for age, sex, civil status, education, occupation, income, residence, and type of insurance. Significance testing was done through calculating the 95% confidence intervals and a *P* value less than 0.05. Data analysis was performed using SPSS version 26 (SPSS Inc; Chicago, IL, USA).

### Ethical considerations

This research was granted ethical and survey clearances by the National Ethics Committee (NEC Code: 2018–013-Tolabing-Literacy) and the Philippine Statistics Authority (DOH-1840), respectively. Each respondent provided informed consent prior to inclusion in the study, and for participants who were under 18 years old at time of interview, consent of the parent or guardian was also secured.

## Results

### Participant characteristics

A total of 2,303 individuals participated in the national health literacy survey, of which 2,146 respondents were able to complete the questions on functional health literacy. Cases with missing data for at least one variable were excluded from the analysis for this study. The final set of data used in the analysis included 1,997 participants (86.7%). Those categorized as having adequate FHL were 62.7%. The results for objective no. 1 is presented in Table 1 (baseline assessment of the FHL level by characteristics of study participants). Majority of the participants are female (74.6%) who are overrepresented in the current study relative to the proportion reported in the 2020 Census of Population and Housing (49.5%) [33], younger than 59 years of age (88.4%), and were married (67.7%) at the time of interview. Residents of Luzon island and living in urban areas comprised 46.0% and 69.7%, respectively. More than half reported no occupation (53.6%) at the time of interview followed by those categorized as having an unskilled manual occupation (elementary, service and sales) at 22.3%. Participants with public health insurance and belonging to the two lowest income categories are 62.3% and 31.5%, respectively.

**Table 1:** Socio-demographic characteristics of participants (*n* = 1,997)

Characteristics	n	%
Sex		
Female	1,489	74.6
Male	508	25.4
Age (years)		
15-34	782	39.2
35-59	981	49.1
60 and above	234	11.7
Island		
Luzon	919	46.0
NCR	271	13.6
Visayas	297	14.9
Mindanao	510	25.5
Residence		
Rural	605	30.3
Urban	1,392	69.7
Civil status		
Single	486	24.3
Live-in/Married	1,352	67.7
Separated/Divorced	32	1.6
Widowed	127	6.4
Education		
Up to elementary graduate	360	18.0
HS level/graduate	833	41.7
At least college graduate	596	29.8
Vocational	208	10.4
Occupation		
None	1,070	53.6
Professional/Managerial/Technical	153	7.7
Unskilled manual	445	22.3
Skilled manual	106	5.3
Domestic service	83	4.2
Agricultural/forestry/fishery	89	4.5
Armed forces & others	51	2.6
Religion		
Catholic	1,574	78.8
Others	426	21.2
Insurance		
None	478	23.9
Private	150	7.5
Public	1,245	62.3
Private & public	124	6.2
Income (PHP^2^)		
< 40,000	311	15.6
40,000 – 59,999	317	15.9
60,000 – 99,999	483	24.2
100,000 – 249,999	658	32.9
≥ 250,000	228	11.4
FHL score		
Inadequate	744	37.3
Adequate^1^	1,253	62.7

^1^ Functional Health Literacy with score of 60%

^2^ PHP Philippine Peso

Table 2 shows the domains of functional health literacy divided by question. More than half of the participants (68.5%) correctly answered the question representing the prose literacy domain (“*Which vaccine(s) can protect against pneumonia*?”). In contrast, less than half of the participants (41.6%) correctly answered the question representing the numeracy domain (“*How many types of vaccines should have been received by a child who is one year old?*”).

**Table 2:** Scores of participants per Functional Health Literacy question (*n* = 1,997)

FHL^1^ question	Correct (n)	%
1. Which vaccines may be given to infants immediately after birth?	1,271	63.6
2. How many types of vaccines should have been received by a child who is one year old?	830	41.6
3. In total, how many doses of all the recommended vaccines should be given to infants?	1,089	54.5
4. Which vaccine/s can protect against pneumonia?	1,368	68.5
5. Assume that you have a child who was born on January 1, 2017, when should he/she receive the first dose of the measles-mumps-rubella (MMR) vaccine?	1,240	62.1

^1^ Functional Health Literacy

### Association between functional health literacy and participant characteristics

Table 3 presents the association between FHL adequacy and socio-demographic characteristics of the participants in response to objective no. 2. After adjustment for socio-demographic and economic factors, a significant association between FHL adequacy and education was found, demonstrating that individuals with a college level education are more likely to exhibit adequate FHL (Adjusted prevalence ratio [APR]: 1.43, 95% CI 1.27–1.60). Participants with either a live-in residing in urban areas (APR: 1.14, 95% CI 1.06–1.24), in the highest income group (APR: 1.29, 95% CI 1.14–1.47), and with public health insurance (APR: 1.11, 95% CI 1.01–1.21) are substantially more prevalent of exhibiting FHL adequacy compared to participants residing in rural areas, belonging to the lowest income group, and without health insurance, respectively. Participants categorized under the domestic service occupation (e.g.: students/housewives/retirees) [34, 35] (APR: 1.19, 95% CI 1.03–1.37) are significantly more likely to demonstrate FHL adequacy compared to those with no occupation. Those who are married or with a live-in partner likewise presents a higher likelihood of exhibiting FHL adequacy though significance was not observed.

**Table 3:** Adjusted prevalence ratios of FHL adequacy and socio-demographic characteristics of participants (*n* = 1,997)

Characteristics	APR^1^ (95% CI^2^)	*P*^3^
Sex		
Female	Ref 0.92 (0.84–0.99)	
Male		0.03
Age (years)		
15–35	Ref	
36–59	0.85 (0.79–0.91)	< 0.001
60 or above	0.72 (0.63–0.82)	< 0.001
Civil status		
Single	Ref	
Live-in/Married	1.01 (0.94–1.10)	0.71
Separated/Divorced	0.79 (0.56–1.13)	0.19
Widowed	0.79 (0.65–0.95)	0.01
Education		
Up to elementary graduate	Ref	
HS level/graduate	1.23 (1.09–1.38)	0.001
At least college level	1.43 (1.27–1.60)	< 0.001
Vocational	1.46 (1.28–1.67)	< 0.001
Occupation		
None	Ref	
Professional/ managerial/ technical	1.11 (0.98–1.25)	0.11
Unskilled manual	1.06 (0.97–1.15)	0.19
Skilled manual	1.05 (0.91–1,22)	0.50
Domestic service	1.19 (1.03–1.37)	0.02
Agricultural/ forestry/ fishery	0.87 (0.71–1.06)	0.17
Armed forces & others	1.16 (0.96–1.39)	0.12
Income (PHP^4^)		
< 40,000	Ref	
40,000 – 59,999	0.95 (0.83–1.10)	0.51
60,000 – 99,999	1.12 (0.99–1.26)	0.07
100,000 – 249,999	1.17 (1.04–1.30)	0.007
≥ 250,000	1.29 (1.14–1.47)	< 0.001
Residence		
Rural	Ref	
Urban	1.14 (1.06–1.24)	0.001
Insurance		
None	Ref	
Private	1.16 (1.01–1.33)	0.04
Public	1.11 (1.01–1.21)	0.04
Private & public	1.21 (1.15–1.39)	0.009

^1^ Adjusted prevalence ratio

^2^ Confidence interval

^3^ *P* value

^4^ Philippine Peso

In contrast, the adjusted regression results suggest that those who were male (APR 0.92, 95% CI 0.84–0.99) and 60 years or above (APR 0.72, 95% CI 0.63–0.82) presented with lesser likelihood of demonstrating adequate FHL as compared to females and those belonging to the 15 to 35 age group, respectively. Those who are widowed are likewise significantly less likely to have adequate FHL (APR 0.79, 95% CI 0.65–0.95).

## Discussion

The results indicate that more than half of the participants showed adequate FHL in almost all of the questions except for an item representing the numeracy domain. There is a significant association between FHL adequacy and education. Moreover, participants with a partner, residing in urban areas, in the highest income group, and with public health insurance are more likely to show FHL adequacy. Those who are male and 60 years or above, are less likely to demonstrate adequate FHL. Inferences from these results need to take into account the characteristics of the present sample that is predominantly women, middle-aged, and living in Luzon area.

Numeracy is generally more difficult to achieve than prose literacy [36]. This may be the reason why most participants incorrectly answered the item representing the numeracy domain compared to the items representing prose literacy. Considering that more than half of the survey participants reached only high school level or lower, they may not have the educational training for the numeracy domain. However, innumeracy more than illiteracy is found to account for low health literacy [37]. Thus, the innumeracy level of the participants may account for the inadequate FHL results in this study.

In this survey, FHL adequacy is shown among females, younger adults, those with higher education, those who are married or with a live-in partner, those living in urban areas, those belonging to the highest income level and those with public health insurance. Educational status is found to be the most important determinant of health literacy in almost all the studies reviewed about the topic [28, 29, 38]. It is logical that the knowledge and skills gained in the higher levels of education equips the person with the ability to interpret informational materials adequately. It is therefore important to consider the level of education of the intended audience in designing infographics and other materials to be disseminated. To determine whether the material is understandable, it may be tested to a small group of people with the characteristics of the intended audience. Their feedback is useful in further improving the material. Simplicity and brevity are keys to better understanding, a universal principle that is applicable to everyone, even among the educated.

Age is a factor to consider in literacy. This survey showed that adults 35 years old and above are less likely to have FHL adequacy compared to those aged 15–34 years. Although the present study participants are predominantly middle-aged, this is consistent with the research findings of Shi, et al. [39] wherein those 25–35 years of age have the highest rate of adequate health literacy. It’s worth noting that with increasing age after 34 years, the abilities of a person decline especially among the elderly. In designing informational materials, the content, approach and presentation should be appropriate to the age group.

Females have higher rates of FHL adequacy than males. Although the present study involved significantly more females than males compared to the national census, this is corroborated by several studies [29, 40]. About three-fourths of the participants in this study are female which may account for the higher scores in prose literacy, consistent with prior research findings that women had significantly higher health literacy than men [38]. For the present study, the topic involved vaccinations of children which women may be more concerned about. Participants with either a live-in partner or married are 1.01 times more likely to have FHL adequacy compared to participants who are single. Since married persons have children who need vaccinations, they tend to seek information related to vaccinations, exposing them to vaccination-related materials, thus increasing awareness of how to read and understand the material provided for the FHL questions. Often, they would have paid attention more keenly on the details about vaccination since this involved their own child or children. Domestic service occupation (e.g., housewives) is found to be more likely to demonstrate adequate FHL. This is similar with the foregoing discussions about married persons since housewives are the ones directly taking care of their children. Moreover, having a partner or significant other may have contributed to this higher FHL since being separated or widowed is not significantly associated with FHL adequacy, as presented in the adjusted regression model. Hence, the presence of a significant other influences one’s health-seeking behaviors as insinuated in the study of Xu, et al. [41] about one’s vaccine hesitancy being influenced by one’s spouse.

High income groups are almost twice as likely to have FHL adequacy than low-income groups. Prior research also showed that the higher the income, the higher rate of adequate health literacy [29, 36]. Having more money gives one an advantage over education, experiences, and other opportunities in life. Similarly, participants residing in urban areas showed FHL adequacy, both in the unadjusted and adjusted models. This is consistent with the research findings of Shi, et. al. [39]. Urban living exposes residents to more informational opportunities than those in the rural areas, thus giving them continuous exposure to communication media. Moreover, healthcare facilities are more physically accessible to their residence and/or workplaces, providing more healthcare opportunities. For instance, rural dwellers may need to travel for hours to reach the nearest healthcare center or talk with a healthcare worker. Hence, any encounter with rural residents should be maximized for health learnings. In addition to one-on-one health teachings, they should be provided with materials that they can refer to when they are on their own in the hinterlands.

Respondents with public insurance have a higher rate of FHL adequacy in this survey. The public insurance includes vaccinations among others, which private insurances do not usually cover. Moreover, those who avail of insurance are more likely to be conscientious of their health. Thus, they have better abilities to navigate through the health information provided to them.

One limitation of this study is the low sensitivity of the instrument, although it had a high specificity of 97%. This indicated that some persons with inadequate FHL may be misclassified as having adequate FHL, and therefore the reported proportion with adequate FHL may be an overestimate of the actual proportion. This article only focused on FHL. Another limitation is the greater representation of women compared to men in the study. This may have resulted from the house-to-house recruitment and interviews of participants. Despite best efforts to interview the persons randomly sampled from each household, the call-back protocol of this study resulted in some sampled respondents being dropped after three failed attempts for interview, and women were mostly the ones in the houses during the time of data collection. Because of this non-representativeness, while the correlations reported in this study provide insight to associations of FHL with socioeconomic and demographic characteristics, the prevalence of adequate FHL in this study may not be representative of the overall prevalence in the country [42].

## Conclusions

Most Filipinos have higher prose literacy than numeracy, and more than half of the respondents exhibit FHL adequacy. Males, older adults, single, lower education, low income, and rural dwellers, and possibly males, have higher risk for FHL inadequacy. Moreover, having public health insurance may have helped in raising one’s FHL through increased access to health services, thus providing more learning opportunities during client-provider interactions and in navigating the healthcare system.

These findings have implications in the provision of health information to patients and communities which comprise the following study recommendations: Clearer and simpler language should be used in IECs, with no or less quantitative data for those with higher risk for FHL inadequacy, and the application of other statistical tests such as the log binomial regression analysis for future studies. On the community level, stratifying the target audience according to the characteristics found relevant in this study serves as a basis for the content and design of informational materials for health. Health policies should take into consideration these pertinent socioeconomic and demographic status for population-based educational programs. Interventions to improve functional health literacy should be targeted towards older people, single, with low education and low income living in the rural areas.

## Data Availability

The data that support the findings of this study are available from the Philippine Council for Health Research and Development but restrictions apply to the availability of these data, which were used under license for the current study, and so are not publicly available. Data are however available upon reasonable request to klsiongco@up.edu.ph and with permission of the Philippine Council for Health Research and Development.

## Abbreviations

FHL: Functional health literacy
NAAL: National assessment of adult literacy
TOFHLA: Test of functional health literacy in adults
NVS: Newest vital sign
WHO: World Health Organization
CAPI: Computer-assisted personal interview
FHL-5 Test: Functional health literacy-5 test
APR: Adjusted prevalence ratio
CI: Confidence interval
PHP: Philippine Peso
